# Response to Ipilimumab/Nivolumab Rechallenge and BRAF Inhibitor/MEK Inhibitor Rechallenge in a Patient with Advanced Metastatic Melanoma Previously Treated with BRAF Targeted Therapy and Immunotherapy

**DOI:** 10.1155/2020/4392562

**Published:** 2020-06-25

**Authors:** Caitlyn N. Myrdal, Srinath Sundararajan

**Affiliations:** ^1^University of Arizona College of Medicine, Tucson, AZ, USA; ^2^Hematology and Oncology, Texas Oncology, Houston, TX, USA

## Abstract

Little is known about the optimal sequencing of targeted therapy and immunotherapy in the treatment of patients with BRAF^V600^-mutated metastatic melanoma. BRAF/MEK inhibition often has the benefit of rapid disease regression; however, resistance is frequently seen with long-term use. Treatment with immune checkpoint inhibitors offers the potential for long-term response but displays a lower rate of objective response. The benefit of synergy between therapies is apparent; however, there is limited data regarding optimal sequencing in the treatment of advanced melanoma. We present the case of a 62-year-old gentleman with advanced BRAF^V600^-mutated melanoma who followed an unconventional treatment path. After progressing on single-agent vemurafenib, he had response to multiple modalities of immunotherapy before progression. After, he had a substantial response to multiple BRAF/MEK inhibitor rechallenges before developing resistance. The patient is now stable after a retrial of combination immunotherapy. Our case illustrates that with the right sequencing of therapy, meaningful clinical responses can be elicited with rechallenging of targeted therapy and immunotherapy in metastatic melanoma.

## 1. Introduction

Invasive melanoma accounts for roughly 1% of skin cancer cases, although it is responsible for the majority of skin cancer deaths. It is estimated that in 2020, there will be over 100,000 new cases of melanoma and around 7,000 melanoma-related deaths in the United States [1]. BRAF mutation is a targetable genetic alteration seen in about 40-50% of patients with cutaneous melanoma [2, 3]. Based on 2019 NCCN guidelines, the initial treatment of stage I-III melanoma includes wide excision of primary tumor with sentinel lymph node dissection. Systemic options for adjuvant treatment of stage III melanoma include immune checkpoint inhibitors ipilimumab, nivolumab, or pembrolizumab or targeted combination therapy with dabrafenib/trametinib. In patients with metastatic or unresectable disease, guidelines recommend first-line systemic immunotherapy with single-agent anti-PD-1 therapy (pembrolizumab or nivolumab) or combination anti-PD-1 and anti-CTLA-4 agent therapy with nivolumab and ipilimumab. In patients with BRAF^V600^-positive melanoma, combination BRAF and MEK targeted therapy (dabrafenib/trametinib, vemurafenib/cobimetinib, or encorafenib/binimetinib) is an additional option. Second-line therapy decisions are typically based on performance status, prior treatment given, and presence or absence of BRAF domain mutation [4].

As outlined in NCCN guidelines, both immunotherapy and combination targeted therapy are commonly used in the treatment of advanced melanoma. Immune checkpoint inhibitors such as ipilimumab (anticytotoxic T-lymphocyte-associated protein 4 (CTLA-4) antibody), pembrolizumab (antiprogrammed cell death protein 1 (PD-1) antibody), and nivolumab (anti-PD-1 antibody) have been shown to improve survival in patients with advanced melanoma [5–7]. Immunotherapy has demonstrated a lower rate of objective response but has the potential for long-lasting responses [8, 9]. Combined BRAF and MEK inhibitors such as dabrafenib/trametinib, vemurafenib/cobimetinib, and encorafenib/binimetinib also show efficacy in the treatment of patients with advanced BRAF^V600^-mutated melanoma [10–12]. Generally, BRAF/MEK inhibitor therapy exhibits more rapid disease regression but patients often develop resistance, limiting long-term use [13]. The unique advantages and disadvantages of each treatment highlight potential for synergy. Preclinical studies show promise that MAP kinase pathway modulation may create a more favorable tumor microenvironment for immune checkpoint inhibitors [14, 15]. However, clinical trials are lacking and there is still little agreement on optimal sequencing for use of targeted therapy and immunotherapy in the treatment of advanced melanoma.

## 2. Case Presentation

We present the case of a 62-year-old male with advanced melanoma who followed an unconventional treatment path (see Table 1). The patient was diagnosed with stage III BRAF^V600E^ LDH normal melanoma of the right chest in June of 2011. Upon diagnosis, 4 axillary lymph nodes were positive on axillary lymph node dissection. The patient initially decided to forego treatment; however, imaging 9 months later showed numerous subcutaneous and pulmonary metastases. The patient began treatment with BRAF inhibitor vemurafenib in April of 2012. Subsequent serial PET/CT scans two months later indicated mixed response to therapy, with resolution of pulmonary and numerous subcutaneous nodules with the development of multiple new nodal and subcutaneous lesions. Throughout the following two years of vemurafenib therapy, this pattern of new and resolving nodal and subcutaneous metastases continued. Due to an overall decrease in disease burden and patient preference, treatment was continued. In September of 2014, vemurafenib was discontinued due to CT and MRI indicating significant metastases in the brain and bones. The patient completed a course of radiotherapy to L4 and L5 lesions as well as radiosurgical and radiotherapy treatment for the brain metastasis with good response. He was then started on ipilimumab for systemic therapy. After 4 cycles, CT and MRI demonstrated stable disease. Immunotherapy was held due to a period of colitis, but on resolution, a maintenance dose of ipilimumab was given. However, due to significant cutaneous metastasis, ipilimumab was discontinued in late May of 2015, and the decision was made to proceed with wide excision of the subcutaneous masses and hold systemic therapy with reimaging in 6 weeks. At that time, there were no new cutaneous metastases and intracranial/osseous disease was stable. Follow-up imaging was scheduled for another 6 weeks, which again showed stable disease even with continued hold of systemic therapy. At this point, follow-up MRI brain and CT abdomen/pelvis were scheduled for 3 months later.

Follow-up imaging was not completed until January of 2016 but showed stable intracranial and osseous disease with multiple new cutaneous metastases. The lesions were excised, and systemic treatment options were discussed at a tumor board. The patient was lost to follow-up until mid-June 2016, where repeat CT indicated significant progression of cutaneous metastasis. Given continued cutaneous progression, single-agent pembrolizumab was started in June of 2016. The patient had an initial mixed response to pembrolizumab with overall stable disease and remained on the therapy for 9 months. Subsequent PET/CT scan in March of 2017 showed an increased number of pulmonary nodules and approximately 7 new subcutaneous lesions on the patient's legs bilaterally, and pembrolizumab was discontinued.

After discussion, ipilimumab/nivolumab combination therapy was started in April of 2017. After 4 cycles, a PET/CT demonstrated stable disease with the exception of a new subcutaneous metastasis on the ankle. Given the history of extensive treatment and lack of significant progression, single-agent nivolumab was continued and the lesion was treated palliatively with radiation therapy. Imaging in October of 2017 indicated a mixed response, with stable visceral disease but progression in the form of multiple new subcutaneous metastases in the back and right thigh. Lack of open slots in appropriate clinical trials led to continued therapy with nivolumab beyond progression. However, after imaging in December of 2017 showed continued subcutaneous progression, single-agent nivolumab was discontinued. The patient was then started on a clinical trial with intralesional SD-101+systemic pembrolizumab from January 2018 to March 2018 until progression.

**Figure 1:**
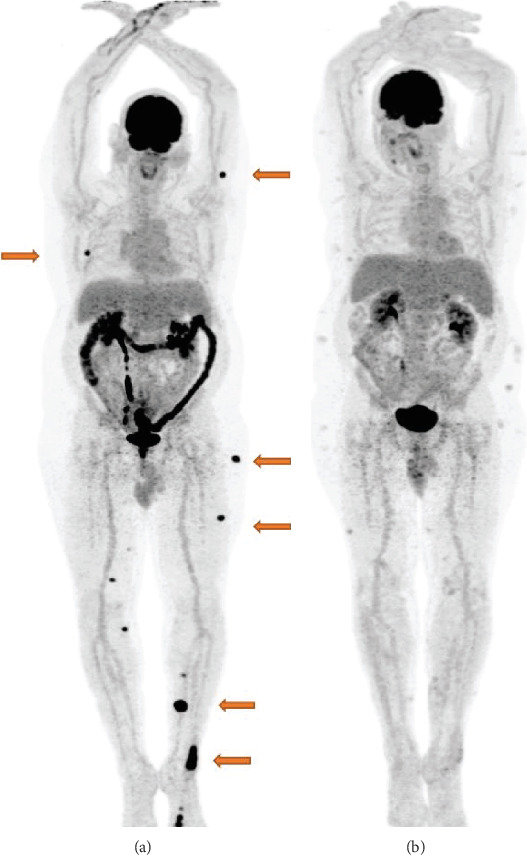
(a) PET/CT scan prior to start of dabrafenib/trametinib combination therapy showing multiple subcutaneous metastases. (b) PET/CT scan 4 months after initiation of BRAFi/MEKi therapy showing resolution of previously noted subcutaneous metastasis.

Since it had been over 3 years since the patient trialed BRAF inhibitor therapy and the patient had never been treated with BRAF inhibitor/MEK inhibitor combination therapy, dabrafenib/trametinib was initiated. The patient first began dabrafenib and trametinib in March of 2018 and had an impressive response with substantial shrinkage of subcutaneous lesions within a few days. In July of 2018, PET/CT showed near-complete response to treatment, with resolution of the pulmonary and subcutaneous nodules (Figure 1). Several areas of hypermetabolic subcutaneous infiltration were seen throughout the body consistent with an inflammatory dermatologic reaction. Response persisted until unfortunately in November of 2018, CT showed evidence of progression with new nodal and soft tissue lesions as well as a single hepatic lesion concerning for metastasis. Dabrafenib/trametinib combination therapy was discontinued.

Given the fact that the patient progressed on all standard lines of treatment and there was no eligible clinical trial available at the time, we decided to proceed with encorafenib/binimetinib combination therapy. The patient was on encorafenib/binimetinib from December 2018 through April 2019 with partial response, until unfortunately imaging indicated progressive disease with new left external iliac nodal metastasis and soft tissue deposits.

**Figure 2:**
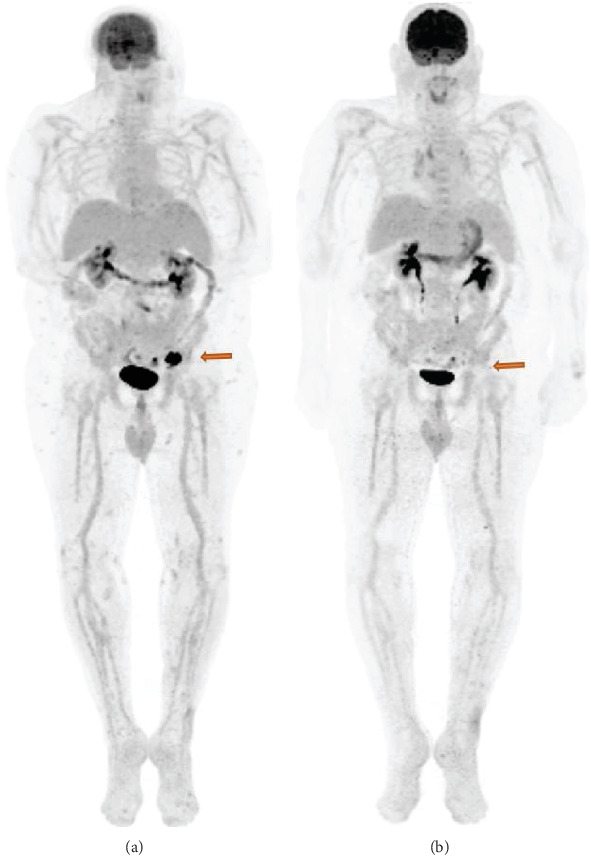
(a) PET/CT scan prior to start of ipilimumab/nivolumab immunotherapy showing FDG avid 2.8 × 3.9 cm left inguinal lymphadenopathy. (b) PET/CT scan 4 months after reinitiation of ipilimumab/nivolumab immunotherapy showing near-complete resolution of the left iliac lymphadenopathy.

As the patient had exhausted and progressed on all standard lines of treatment and no clinical trial was available for him at the time, we discussed potential treatment with nab-paclitaxel chemotherapy or retrial of ipilimumab/nivolumab immunotherapy. The patient had shown response to ipilimumab/nivolumab therapy in the past, with progression in the maintenance phase. This context was paired with data from studies suggesting the potential for immunotherapy after BRAF inhibition owing to favorable modulation of tumor microenvironment [14, 15]. The patient decided to proceed with combination ipilimumab/nivolumab immunotherapy and began treatment in April of 2019. PET/CT in late-July 2019 showed mixed response to therapy, with a significant decrease in nodal and subcutaneous FDG avidity with a stable small left hepatic lobe lesion that was previously noted in November 2018 (Figure 2). After 5 treatment cycles, CT imaging in September 2019 showed a decrease in nodal metastasis size, a stable hepatic lesion, and no evidence of pulmonary disease. CT imaging completed 11/2019 showed no change in the nodal or hepatic lesion, indicating stable disease.

## 3. Discussion

Treatment of metastatic melanoma has significantly advanced with the introduction of immunotherapy and continued developments in targeted therapy. Targeted therapies that work at the level of the MAP kinase pathway, such as BRAF and MEK inhibitors, show a substantial initial response in decreasing tumor burden [16]. Combination of BRAF and MEK inhibitors enhances response rates and extends progression-free survival [10–12]. However, even with combination, treatment tends to display a limited duration of response with most patients eventually displaying progression [13]. Immune checkpoint inhibitors, such as anti-CTLA-4, PD-1, and PD-L1 antibodies, have shown clear effectiveness in treatment of metastatic melanoma [5–7]. Although immunotherapy may have a slower onset of action and lower rate of objective response, long-term follow-up has demonstrated that responses can be lasting [8, 9]. Thus, immunotherapy may have a more modest initial response but is thought to offer the advantage of durable disease control.

With each treatment modality posing unique advantages and disadvantages, there may be a potential benefit of synergy. What if the response rates of targeted therapy could be combined with the lasting effects of immunotherapy? Multiple early preclinical studies have examined this idea, finding that BRAF/MEK inhibitor therapy increases expression of melanoma antigen presentation and tumor CD8+ T-cell infiltrate while decreasing immunosuppressive cytokines [15]. Interestingly, BRAF inhibition was also found to increase expression of immunosuppressive ligand PD-L1 [14, 15]. These findings suggest that immune checkpoint inhibition after targeted therapy may be crucial to reverse BRAF/MEK-induced immune escape in melanoma. Although this data highlights potential for synergy in the preclinical setting, there is continued discussion on optimal sequencing of therapy.

One idea is that immunotherapy and targeted therapy could be administered concomitantly. This is based on the finding that T-cell infiltrate and melanoma antigen expression decreased at the time of BRAF/MEK inhibitor resistance [15]. If this is the case, immune advantages of MAP kinase modulation would be lost shortly after disease progression. Furthermore, it may be argued that in this process, the tumor gains additional immunosuppressive mechanisms, possibly leading to subsequent failure on immunotherapy. Interestingly, a recent report investigating solutions to BRAF inhibitor resistance found that intermittent dosing may delay the onset of drug-resistant disease [17]. If the benefit of immunotherapy in the context of BRAF/MEK inhibitor tumor microenvironment modulation is confined to a window, strategies to delay resistance could potentially allow more time for immunotherapy response after BRAF/MEK inhibition.

Clinical trials examining potential synergy between treatments have been lacking. Early trials combining ipilimumab and vemurafenib revealed unexpected hepatotoxic toxicity, leading to a discontinuation of the study [18]. Further dose-adjusted studies yielded conflicting results. One retrospective study showed no statistical significance in response rate, overall or progressive free survival based on whether patients first received immunotherapy or a BRAF inhibitor [19]. Similar findings were found in another retrospective report, indicating a similar rate of overall survival irrespective of whether patients received a BRAF or MEK inhibitor or an anti-PD-1 agent first. However, those who benefited from BRAF inhibition for over 6 months showed significantly higher response to anti-PD-1 therapy [20]. Conversely, a retrospective report found that longer overall survival was observed when ipilimumab was administered prior to a BRAF inhibitor compared with a BRAF inhibitor followed by ipilimumab or either alone [21]. An exciting recent randomized phase 2 trial found that triplet therapy with dabrafenib, trametinib, and pembrolizumab extended progression-free survival and duration of response around 6 months compared to doublet therapy with dabrafenib and trametinib [22].

## 4. Conclusion

Optimal sequencing of BRAF/MEK inhibitors and immunotherapy in the treatment of metastatic melanoma is still controversial. We report a patient who had an unconventional timeline of treatment with both therapies. After progressing on single-agent vemurafenib, there was response to multiple modalities of immunotherapy. After, he had a substantial response to dabrafenib/trametinib before developing resistance. Despite just failing dabrafenib/trametinib therapy, the patient then had a clinically meaningful disease stability with subsequent encorafenib/binimetinib treatment before progression. The patient is currently doing well after a retrial of combination immunotherapy with ipilimumab/nivolumab over 8 months. This case may provide context in the ongoing puzzle, suggesting utility of immunotherapy retrial after progression on multiple modalities of BRAF/MEK inhibition. However, this data is limited to a single case report, and findings here need validation in a large sample size prospectively. Additionally, it is important to recognize that disease burden plays a significant role on which drug to administer first, as targeted therapy may be a better option for patients with rapidly progressing disease. Further randomized clinical trials examining the synergy between targeted therapy and immunotherapy are greatly needed to compound the respective advantages of each and provide more robust and lasting responses to treatment.

## Figures and Tables

**Table 1:** Timeline of treatment.

Treatment	Dose and frequency	Timeline (month/year)	Reason for cessation	Months of therapy
Vemurafenib	960 mg (4 × 240 mg) orally twice daily (BID)	4/2012–9/2014	Progression	29
Ipilimumab	4 cycles induction q3 weeks at 3 mg/kg, followed by maintenance treatment	9/2014–5/2015	Progression	8
Pembrolizumab	2 mg/kg q3 weeks, completed 11 cycles	6/2016–3/2017	Progression	9
Ipilimumab/nivolumab	4 cycles induction ipilimumab 3 mg/kg+nivolumab 1 mg/kg q3 weeks, 2 cycles maintenance nivolumab 240 mg q2 weeks	4/2017–12/2017	Progression	8
Intralesional SD-101+pembrolizumab (study)	8 mg SD-101 injection, pembrolizumab 200 mg q3 weeks	1/2018–3/2018	Progression	3
Dabrafenib/trametinib	Dabrafenib 150 mg BID initially, dose reduced 75 mg BID, trametinib 2 mg daily	3/2018–11/2018	Progression	8
Encorafenib/binimetinib	Encorafenib 450 mg daily, binimetinib 45 mg BID	12/2018–4/2019	Progression	4
Ipilimumab/nivolumab retreatment	4 cycles induction 3 mg/kg ipilimumab+1 mg/kg nivolumab q3 weeks, 5 cycles maintenance nivolumab 480mg q4 weeks	4/2019–present (12/2019)		Ongoing treatment for 8 months
